# Abstinence from repeat revascularization may suggest poor outcome

**DOI:** 10.1093/ejcts/ezaf046

**Published:** 2025-02-17

**Authors:** Ari Mennander

**Affiliations:** Faculty of Medicine and Health Technology, Tampere University, Tampere, Finland; Tampere University Hospital, Heart Hospital, Tampere, Finland

**Keywords:** Coronary artery bypass grafting, Diabetes mellitus, Repeat revascularization

Though diabetes mellitus (DM) is a chronic disease and a risk factor for coronary artery disease, the long-term outcome of patients with DM after coronary artery bypass grafting (CABG) is unclear. DM impacts not only the coronary arteries of the heart but also the intramyocardial arteries. Despite successful surgery and revascularization of the main coronary artery regions during CABG, ongoing intramyocardial arteriosclerosis may eventually lead to reduced outflow in revascularized coronary artery regions [1, 2].

In the current study by Barili *et al.* [3], the outcome of patients with and without DM is compared after CABG. This is an observational cohort investigation merging 2 multicentre studies conducted in 2002–2004 and 2007–2008 on isolated CABG on almost 11 000 patients encompassing 3545 patients with DM. The primary outcome was a composite Major Adverse Cardiac and Cerebrovascular Event (MACCE). The analyses were adjusted for baseline differences using propensity scores for inverse probability of treatment weight.

DM did not affect short-term mortality and repeat revascularization but was associated with lower incidence of 30-day MACCE, myocardial infarction and stroke. At 10 years of follow-up, patients with DM experienced increased risk of MACCE, mortality, stroke and myocardial infarction as compared to those without DM. Interestingly, there was no association of repeat revascularization and myocardial infarction during follow-up.

The main message of the well-written manuscript is the association between long-term outcomes with DM after CABG. The presented results are delicious to digest. The relatively moderate number of repeat revascularization in patients with or without DM may be flattering to surgeons. Decision-making for CABG seems justified for many patients with DM, though the chronic nature of the disease also signifies an ongoing increased risk of MACCE, mortality, stroke and myocardial infarction. Indeed, in a previous study, repeat revascularization was more frequent after percutaneous coronary intervention (PCI) versus CABG irrespective of DM, whereas mortality, myocardial ischaemia and repeat revascularization were higher in patients with DM undergoing PCI versus CABG as compared to those without DM during a median follow-up of 11.8 years [4].

Statistically, the proportional hazards assumption was not met in the current study [3]. The outcome curves for MACCE and mortality seem to diverge considerably but only after the early postoperative period. This is reflected in the surprisingly better early outcome of MACCE, myocardial infarction and stroke in patients with DM as compared with those without. There seems to be 2 different time windows of the impact of diabetes on outcome, the perioperative phase and the long-term follow-up time. A plausible explanation may be related to the retrospective nature of the study, including only the patients who underwent CABG. The 1-year outcome after CABG in patients with versus without DM should not differ [5, 6]. In general, a reverse myocardial perfusion pattern after myocardial ischaemia may be expected in patients with or without DM after successful CABG [6]. It would be interesting to compare the results of this study to those obtained after PCI in patients with and without DM and to balance the results with the time of onset of DM.

Many matters are worth discussing when comparing patients with and without DM. As an entity of many chronic diseases, DM may not be considered similar in every patient. Different oral and insulin medications, least to mention medication compliance of any treatment, and periods of hypo- and hyperglycaemia, may interfere with outcome. The selection and number of bypass grafts of arterial and venous origin may differ in patients. Due to the ongoing disease nature of DM, repeat revascularization may not have been considered technically feasible in some patients despite ongoing myocardial ischaemia and infarction; decreased number of repeat revascularization may, unfortunately, also signify increased mortality in the comorbid patient with, e.g. DM.

The clinical presence and significance of ongoing intramyocardial arteriosclerosis in patients with DM deserves further attention. The patients included in this study [3] were treated some 20 years ago, and current treatment protocols may differ substantially including medication. Meanwhile, the clinical data confirms the impact of DM on long-term outcome after CABG.
